# Influence of Survivorship Care on Health‐Related Quality of Life, Knowledge of Late Effects, and Distress Levels Among Long‐Term Hodgkin Lymphoma Survivors

**DOI:** 10.1002/cam4.71113

**Published:** 2025-08-05

**Authors:** Eline M. J. Lammers, Annelies Nijdam, Michael Schaapveld, Lonneke V. van de Poll‐Franse, Cécile P. M. Janus, Karin M. Aarsman, Liane C. J. te Boome, Nathalie B. Tomasoa, Rinske S. Boersma, Saskia E. Rademakers, Floriske G. Stedema, Lara H. Böhmer, Marieke van den Berg, Adriaantje C. Kroeze, Leonie S. Strobbe, Wendy Deenik, Heleen S. de Lil, Flora E. van Leeuwen, Berthe M. P. Aleman, Josée M. Zijlstra, the BETER consortium

**Affiliations:** ^1^ Department of Psychosocial Research & Epidemiology The Netherlands Cancer Institute Amsterdam the Netherlands; ^2^ Department of Medical and Clinical Psychology Tilburg University Tilburg the Netherlands; ^3^ Department of Radiation Oncology Erasmus University Medical Center Rotterdam the Netherlands; ^4^ Department of Hematology University Medical Center Utrecht Utrecht the Netherlands; ^5^ Department of Hematology Haaglanden Medical Center, Location Antoniushove Leidschendam the Netherlands; ^6^ Department of Radiation Oncology Institute Verbeeten Tilburg the Netherlands; ^7^ Department of Hematology Amphia Hospital Breda the Netherlands; ^8^ Department of Radiation Oncology Leiden University Medical Center Leiden the Netherlands; ^9^ Department of Hematology University Medical Center Groningen Groningen the Netherlands; ^10^ Department of Hematology Haga Hospital The Hague the Netherlands; ^11^ Department of Hematology Catharina Hospital Eindhoven the Netherlands; ^12^ Department of Hematology Meander Medical Center Amersfoort the Netherlands; ^13^ Department of Hematology Gelre Hospitals Zutphen and Apeldoorn the Netherlands; ^14^ Department of Hematology Rijnstate Hospital Arnhem the Netherlands; ^15^ Department of Hematology Maxima Medical Center Eindhoven and Veldhoven the Netherlands; ^16^ Department of Radiation Oncology The Netherlands Cancer Institute Amsterdam the Netherlands; ^17^ Department of Hematology Amsterdam UMC, Location Vrije Universiteit, Cancer Center Amsterdam Amsterdam the Netherlands

**Keywords:** distress, health‐related quality of life, Hodgkin lymphoma, knowledge, patient‐reported outcomes, survivorship care, worries

## Abstract

**Introduction:**

Hodgkin lymphoma (HL) is a highly curable malignancy mainly affecting young adults, but survivors are at risk of serious late adverse effects, like cardiovascular disease and subsequent malignancies, which may impact health‐related quality of life (HRQoL). The Dutch BETER consortium introduced a nationwide survivorship care program offering tailored screening for late effects among HL survivors from 5 years after diagnosis onward. This study evaluates the association between BETER survivorship care and HRQoL, knowledge of late effects, and distress levels.

**Methods:**

The INSIGHT study is a nationwide retrospective cohort study comparing HL survivors who received BETER care since 2013‐2016 to matched survivors who did not receive survivorship care until 2019–2024. HRQoL was cross‐sectionally assessed in 2021–2024 (median time since HL diagnosis ~25 years) using the SF‐36 and EQ‐5D‐5L questionnaires. Knowledge of late effects and distress levels were evaluated using a modified Cancer Worry Scale and a 15‐item knowledge questionnaire. Multivariable negative binomial regression analyses were performed to compare outcomes between groups.

**Results:**

HRQoL outcomes showed no significant differences between survivors screened since 2013‐2016 (*n* = 251) and the comparison group (*n* = 119). Overall, HRQoL of HL survivors matched that of the general population in the Netherlands. Knowledge of late effects was suboptimal in both groups, and distress levels were similarly low. Eighty percent of survivors perceived BETER care as beneficial, with most individuals stating that increased knowledge outweighed potential worries.

**Conclusion:**

BETER survivorship care was not associated with better HRQoL among long‐term HL survivors, but the care was perceived as beneficial by survivors. Future (qualitative) research could focus on survivors' preferences for education about their risk of late effects.

## 1 Introduction

Hodgkin lymphoma (HL) is a highly curable malignancy, mainly affecting young people between 20 and 40 years of age [1, 2]. HL survivors are at increased risk of various late effects of treatment, including cardiovascular diseases, subsequent malignancies, hypothyroidism, and severe infections [3, 4, 5, 6, 7]. Some studies show that HL survivors experience a lower health‐related quality of life (HRQoL) than the general population [8, 9].

The Dutch BETER (better care after lymphoma, evaluation of long‐term treatment effects and screening recommendations) consortium has developed a nationwide infrastructure of survivorship care clinics where HL survivors receive personalized screening for (risk factors for) late adverse events according to nationally approved guidelines, from five years after diagnosis onwards [10, 11]. BETER survivorship care was set up with the aims to reduce morbidity and mortality from adverse effects of lymphoma treatment and to improve survivors' quality of life. Screening procedures and schedules are based on survivor and treatment characteristics (e.g., sex, age, radiotherapy (RT) fields, chemotherapy (CT) type and dose, and whether or not splenectomy was performed). Between 2013 and 2016, the first BETER clinics were established, and in the following years, BETER care was gradually implemented in other lymphoma treatment centers across the Netherlands. BETER care is provided by hematologists, radiation oncologists, and nurse specialists, in collaboration with other specialists such as cardiologists and general practitioners.

Few studies have evaluated the effects of receiving cancer survivorship care on HRQoL. To date, one study evaluated the association between survivorship care clinic attendance and HRQoL in adult lymphoma survivors (among which a small group of HL survivors) [12]. This study, conducted in the United States, showed no overall difference in HRQoL between attenders and non‐attenders of survivorship care. Other studies, which almost exclusively focused on survivorship care for childhood cancer and breast cancer survivors, did not find an association between structured survivorship care and HRQoL either; most studies measured HRQoL after a short follow‐up time [13, 14, 15, 16, 17].

Survivorship care has previously been shown to increase survivors' knowledge of their cancer treatment and risk of late adverse effects, probably making survivors better aware of possible symptoms and enabling them to seek appropriate help [12, 16, 18]. Such increased knowledge may, on the other hand, increase survivors' worries about the risk of late adverse effects [15, 16].

In this study, we assessed whether HRQoL differs between HL survivors who received BETER survivorship care over the past decade and those who did not receive (structured) cancer survivorship care until recently. Furthermore, we assessed whether BETER survivorship care affects survivors' knowledge of and distress about late adverse effects. In addition, we evaluated how survivors experienced BETER care, for example, in terms of perceived benefits and burden.

## 2 Methods

### 2.1 Study Population and Design

The INSIGHT study (Improving Nationwide Survivorship care Infrastructure and Guidelines after Hodgkin lymphoma Treatment) is a retrospective cohort study embedded in the national BETER survivorship care infrastructure. The design and rationale of the study were previously published [19]. The study design takes advantage of the gradual implementation of BETER care in the different lymphoma treatment centers in the Netherlands, providing the opportunity to compare survivors who did and did not receive BETER survivorship care over the past 6–10 years.

We compared HRQoL, knowledge, and distress levels between two groups of HL survivors: those who received care at a BETER clinic since 2013‐2016, and those who were eligible for BETER survivorship care during the same period but were not invited until 2019–2024 due to the absence of a BETER clinic at their lymphoma treatment center before that time. The latter group of survivors was frequency matched to the group of survivors who first visited a BETER clinic between 2013 and 2016, based on the following characteristics: sex, age at diagnosis (±5 years), age in 2013 (±5 years), and the following dichotomous (yes/no) treatment variables: chest RT, neck RT, anthracycline‐based CT, and spleen RT or splenectomy.

**FIGURE 1:**
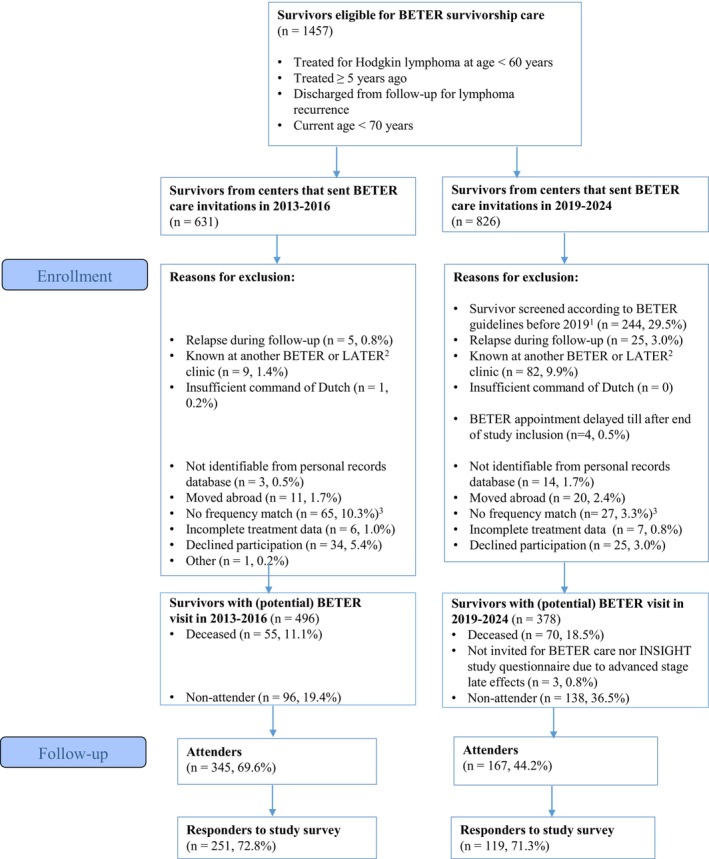
Flow chart summarizing study inclusion. ^1^By study protocol, survivors could not be included in this group when they had received care according to the BETER guidelines before 2019. ^2^LATER is the Dutch survivorship care program for childhood cancer survivors. ^3^These survivors were not selected for inclusion based on the frequency matching criteria (methods, section study population, and design).

HL survivors who have survived at least five years, were treated before the age of 60 years, and are currently not older than 70 years are eligible for BETER survivorship care and for participation in the INSIGHT study [10, 11, 19]. Reasons for exclusion from the study were, among others, inability to be traced in the Dutch Personal Records Database (to verify vital status and current address), living abroad, or decline of informed consent (Figure 1). Survivor enrollment and assessment of patient‐reported outcomes of all included survivors took place in 2021–2024. For logistical reasons, survivors originating from BETER clinics starting in 2019–2024 could only be invited for study participation after their first BETER visit.

The study protocol was approved by the Institutional Review Board of The Netherlands Cancer Institute (IRB21‐115), and we complied with local ethical approval guidelines at all 15 participating centers.

### 2.2 Recruitment and Data Collection

In each participating center, eligible survivors were identified and linked to the Dutch Personal Records Database to verify survivors' vital status and address. Subsequently, eligible survivors who were alive were approached by their treating BETER caregiver through regular mail and were asked to fill out a study survey and informed consent form. In case of non‐response, survivors received two reminders. Survivors could access the survey via a personal link to Castor Electronic Data Capture; a paper version could be supplied upon survivors' request and was included in the second reminder. The study survey included the standardized 5‐dimension/5‐level EuroQol (EQ‐5D‐5L) and 36‐item Short Form Health Survey (SF‐36) questionnaires to assess HRQoL [20, 21]. Furthermore, it comprised the following questionnaires (Tables S1–S3); a modified version of the Cancer Worry Scale (CWS) to assess worries about late adverse effects (the original CWS assesses worries about developing cancer (again)), a 15‐item questionnaire to test knowledge of late adverse effects (including 3 items that only applied to females) and a 5‐item questionnaire to examine whether the survivor: (1) was aware of late adverse effects before the BETER clinic invitation; (2) feels that more knowledge about late effects outweighs possible worries; (3) can talk about possible worries with his/her BETER caregiver; (4) finds BETER care burdensome; and (5) perceives BETER care as beneficial [22].

Survivors were also asked to provide their highest education level: (1) high: University or University of Applied Sciences, (2) intermediate: Secondary Vocational Education and Training, Senior General Secondary Education, or University Preparatory Education, or (3) low: Preparatory Vocational Secondary Education, only primary education, or no education. Socio‐economic status (SES) score was derived based on survivors' zip codes at study inclusion using Statistics Netherlands data (version 2021, excluding students) [23].

Survivor and lymphoma treatment characteristics had been previously collected in all participating BETER centers [11]. HL treatment was categorized based on the associated risk of late effects (especially regarding cardiovascular disease and subsequent malignancies). More specifically, RT was categorized into (1) full mantle field RT; (2) mediastinal RT, excluding full mantle field; (3) RT to neck, excluding mediastinal and full mantle field RT; or (4) RT to other fields or no RT. Chemotherapy was categorized according to the cytostatic drugs administered: (1) both anthracyclines and procarbazine; (2) anthracyclines but no procarbazine; (3) procarbazine but no anthracyclines; or (4) no CT.

### 2.3 Outcomes

To calculate the EQ‐5D‐5L index score, Dutch weights were attached to the levels of each dimension of the questionnaire. Subsequently, the index score for each survivor was derived by deducting the individual score from 1 (= perfect health) [24, 25]. The SF‐36 physical component summary score (PCS) and mental component summary scores (MCS) were calculated according to the corresponding manuals, and using means and standard deviations (SDs) of the Dutch general population for the subscales, as well as Dutch oblique factor score coefficients (i.e., weights) [20, 26, 27, 28]. On the knowledge questionnaire, survivors could score a maximum of three points for each “correctly” answered question (see Table S1). For each survivor, the score on the knowledge questionnaire was calculated as the cumulative number of points divided by the total number of points that could be scored on the questionnaire (45 points for females and 36 points for males), and multiplied by 100.

### 2.4 Statistical Analysis

Missing values for education level (0.01% missing), the EQ‐5D‐5L index score (3.2% missing), the SF‐36 summary scores (18.4% missing for both the PCS and MCS), the modified CWS score (3.2% missing), and the knowledge score (14.1% missing) were handled by multiple imputation using the MICE package in R. Polytomous regression imputation was applied for unordered categorical data and predictive mean matching for continuous covariates, resulting in 20 imputed datasets [29]. Variance inflation factors were calculated to check for multicollinearity. As the scores on the EQ‐5D‐5L index scores, SF‐36 PCS, SF‐36 MCS, modified CWS, and knowledge questionnaire were non‐normally distributed, medians and interquartile ranges (IQRs) were presented, and multivariable negative binomial regression models were used to compare these outcomes between the survivors who started follow‐up at a BETER clinic in 2013–2016 and those who started BETER follow‐up in 2019–2024. All analyses were adjusted for sex, age at diagnosis, age at study inclusion, RT regimen, CT regimen, splenectomy/spleen RT, and education level. Age at study inclusion and age at diagnosis showed non‐linear relationships with the log‐transformed outcomes; therefore, these covariates were categorized as follows: 27–40, 41–55, and 56–71 years for age at inclusion, and 10–25, 26–40, and 41–59 years for age at diagnosis. Estimates resulting from the analyses in the imputed datasets were pooled using Rubin's rule.

As sensitivity analyses, the above described regression analyses were also performed comparing survivors who had visited the BETER clinic at least twice from 2013 to 2016 onwards (in other words excluding those who had dropped out of the BETER program after the first visit), with those who had only visited the BETER clinic once in 2019–2024, before filling in the study questionnaire. For the latter group, we assumed that they had only visited the clinic once when less than a year had passed between their first BETER visit and survey completion, as the BETER guidelines recommend screening at annual, biennial, or five‐yearly time intervals depending on survivor and treatment characteristics [10, 11]. The regression analyses for knowledge and distress about late effects were also performed in males and females separately.

Descriptive statistics (proportions) were used to present the results of the survey on survivors' experience with BETER care. All descriptive data shown refer to the complete cases, unless specified that imputed data is shown. All analyses were performed in RStudio version 1.4.1103.

## 3 Results

### 3.1 Survivor Characteristics and Survey Response Rate

Eleven percent of survivors invited to the BETER clinics between 2013 and 2016, and 18.5% of survivors originating from BETER centers starting between 2019 and 2024, could not be invited for study participation because they had passed away after 2013 (Figure 1). Of the 512 BETER clinic attenders invited to participate in this study, 370 survivors filled in the study questionnaire and were included in the analysis: 251 survivors who first visited a BETER clinic in 2013–2016 and 119 survivors who first visited a BETER clinic in 2019–2024 (Table 1 and Figure 1). The overall response rate was 72.1% and did not differ between the groups. The median age was 55.8 years [IQR: 47.5, 62.4] for responders versus 48.8 years [IQR: 42.3, 54.9] among non‐responders (Table S4).

**TABLE 1:** Patient and treatment characteristics of survivors who attended the BETER clinic and responded to our study questionnaire.

		Survivors followed at a BETER clinic since 2013–2016	Survivors without survivorship care until 2019–2024	*p* ^d^
*n* (%)		251 (67.8)	119 (32.2)	
Female sex (%)		136 (54.2)	59 (49.6)	0.473
Age at HL diagnosis (years) (median [IQR])		24.7 [21.0, 32.3]	29.4 [22.7, 36.9]	0.003
Age at HL diagnosis (years) (cat.) (%)	10–19	48 (19.1)	19 (16.0)	0.036
	20–29	129 (51.4)	47 (39.5)	
	30–39	53 (21.1)	34 (28.6)	
	40–49	19 (7.6)	15 (12.6)	
	50–59	2 (0.8)	4 (3.4)	
Year of HL diagnosis (cat.) (%)	1971–1980	15 (6.0)	7 (5.9)	0.339
	1981–1990	57 (22.7)	18 (15.1)	
	1991–2000	86 (34.3)	49 (41.2)	
	2001–2011	93 (37.1)	45 (37.8)	
Age at survey completion (years) (median [IQR])		55.1 [45.2, 61.9]	57.1 [49.2, 63.2]	0.028
Age at survey completion (years) (cat.) (%)	27–34	7 (2.8)	1 (0.8)	0.043
	35–44	55 (21.9)	12 (10.1)	
	45–54	63 (25.1)	36 (30.3)	
	55–64	96 (38.2)	51 (42.9)	
	65–71	30 (12.0)	19 (16.0)	
Time between diagnosis and survey completion (years) (median [IQR])		24.4 [18.0, 33.2]	25.2 [20.4, 31.1]	0.477
Time between last BETER visit and survey completion (years (median [IQR]))		1.76 [0.62, 5.45]	0.32 [0.11, 0.88]	< 0.001
Age at first BETER visit (years) (median [IQR])		47.6 [38.4, 54.2]	56.4 [48.6, 62.5]	< 0.001
Age at first BETER visit (years) (cat.) (%)	20–29	15 (6.0)	0 (0.0)	< 0.001
	30–39	58 (23.1)	7 (5.9)	
	40–49	70 (27.9)	28 (23.5)	
	50–59	99 (39.4)	44 (37.0)	
	60–70	9 (3.6)	40 (33.6)	
Number of BETER clinic visits before survey administration (%)	1	50 (19.9)	92 (77.3)	< 0.001
	2–4	52 (20.7)	0 (0)	
	5–7	74 (29.5)	0 (0)	
	8–10	65 (25.9)	0 (0)	
	11–16	10 (4.0)	0 (0)	
	Unknown^a^	0 (0)	27 (22.7)	
Time between diagnosis and first BETER visit (years) (median [IQR])		17.3 [10.4, 25.5]	24.6 [19.6, 30.0]	< 0.001
RT regimen (%)	RT to full mantle field	66 (26.3)	26 (21.8)	0.273
	Mediastinal RT but no full mantle field	113 (45.0)	47 (39.5)	
	RT to neck but no mediastinal nor full mantle field RT	21 (8.4)	15 (12.6)	
	RT to other fields or no RT	51 (20.3)	31 (26.1)	
CT regimen (%)	Containing both anthracyclines and procarbazine	80 (31.9)	39 (32.8)	0.445
	Containing anthracyclines but no procarbazine	102 (40.6)	55 (46.2)	
	Containing procarbazine but no anthracyclines	26 (10.4)	12 (10.1)	
	No CT	43 (17.1)	13 (10.9)	
Splenectomy or spleen RT (%)		68 (27.1)	26 (21.8)	0.340
SES score^b^ (median [IQR])		0.18 [0.00, 0.27]	0.17 [0.03, 0.25]	0.848
Highest education level^c^ (%)	High	136 (54.2)	59 (49.6)	0.167
	Intermediate	74 (29.5)	40 (33.6)	
	Low	41 (16.3)	18 (15.1)	
	Unknown (missing)	0 (0.0)	2 (1.7)	

Abbreviations: CT, chemotherapy; HL, Hodgkin lymphoma; RT, radiotherapy; SES, socio‐economic status.

^a^ In general, survivors who first visited a BETER clinic in 2019–2024 filled in the study survey within a year after their first BETER care consultation. For 22.7% of them, the lag time was longer. We have no information on whether these survivors have visited the clinic again in the meantime.

^b^ SES score was based on survivors' zip codes at study inclusion using Statistics Netherlands data (version 2021, excluding students) [23]. Statistics Netherlands derives SES score per zip code from welfare level (i.e., income and assets), education, and recent labor participation. A score of 0 represents the average score in the Netherlands, and a positive/negative score indicates a higher/lower than average SES.

^c^ Education levels were categorized as follows: high: university or university of applied sciences; intermediate: secondary vocational education and training, senior general secondary education or university preparatory education; low: preparatory vocational secondary education, only primary education, or no education.

^d^ Comparisons between the groups were performed using Fisher's exact tests for categorical variables and using Wilcoxon rank‐sum tests for continuous variables.

Fifty‐four percent of survivors who first visited a BETER clinic in 2013–2016 were female vs. 49.6% among those with first visit in 2019–2024. Median age at HL diagnosis was 24.7 years [IQR: 21.0, 32.3] among those who first visited in 2013–2016 versus 29.4 years [IQR: 22.7, 36.9] in the comparison group (Table 1). Seventy‐one percent of survivors from centers that adopted BETER care in 2013–2016 received either full mantle field RT or mediastinal RT compared to 61.3% of survivors in the comparison group.

Age at completion of the study questionnaire was somewhat lower among survivors who first visited the BETER clinic in 2013–2016 compared to those with first visit in 2019–2024 (median 55.1 years [IQR: 45.2, 61.9] and median 57.1 years [IQR: 49.2, 63.2], respectively), while time since HL diagnosis at questionnaire completion was similar (median 24.4 years [IQR: 18.0, 33.2] vs. median 25.2 years [IQR: 20.4, 31.1], respectively). SES score and level of education were also similar in both groups (Table 1). Median time since last BETER visit at study questionnaire completion was 1.76 years [IQR: 0.62, 5.45] for survivors with first BETER visit in 2013–2016 versus 0.32 years [IQR: 0.11, 0.88] in the comparison group.

For survivors from centers that adopted BETER care early, the median age at first BETER visit was 47.6 years [IQR: 38.4, 54.2] versus 56.4 years [IQR: 48.6, 62.5] in the comparison group, and the median time since HL diagnosis was 17.3 years [IQR: 10.4, 25.5] versus 24.6 years [IQR: 19.6, 30.0]. Survivors who started BETER follow‐up in 2013–2016 visited the clinic a median number of six times [IQR: 2, 8] before study survey completion, and 19.9% of them visited the clinic only once (Table 1). The median follow‐up time at the BETER clinic in this group was 6.5 years (IQR: 1.6–8.0, range 0–10.9 years). For those with a first BETER clinic visit in 2019–2024, the last regular follow‐up visit with their treating hematologist or radiation oncologist took place in: 1989–1999 (2.5%), 2000–2009 (9.2%), 2010–2015 (23.5%), 2016–2019 (12.6%), 2020–2024 (16.0%); and for 36.1% there was no date of the last visit available from the medical record.

### 3.2 Health‐Related Quality of Life

**FIGURE 2:**
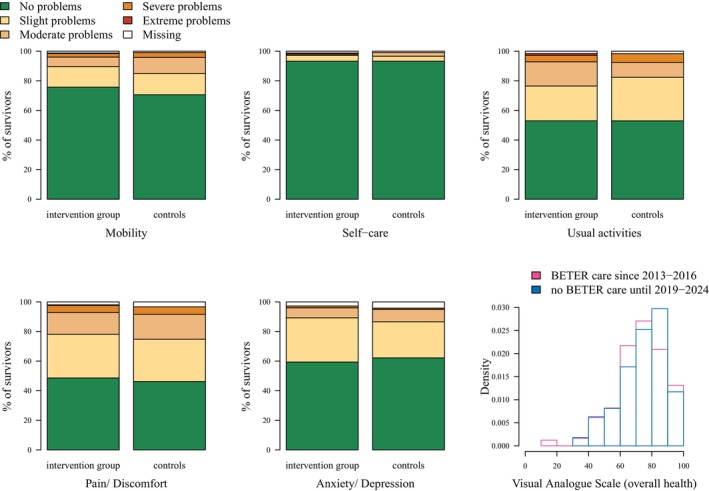
EQ‐5D‐5L subscale scores and a histogram showing the density of the overall health visual analogue scale (VAS) scores in survivors followed at a BETER clinic from 2013–2016 (*n* = 244, excluding 7 missing values) and in those without survivorship care until 2019–2024 (*n* = 111, excluding 8 missing values).

On the EQ‐5D‐5L subscales, disabilities (i.e., any problems) were reported to a similar extent among survivors who started BETER follow‐up in 2013–2016 and those who started in 2019–2024: mobility (23.1% vs. 28.6%), self‐care (5.6% vs. 5.9%), usual activities (both 45.4%), pain/discomfort (49.4% vs. 50.4%), and anxiety/depression (37.8% vs. 33.6%) (Figure 2). The median overall health VAS score was 80 in both groups (IQR: 65.7–86.0 vs. 69.5–86.0). The median EQ‐5D‐5L index score was 0.85 [IQR: 0.77–1.00] for early BETER patients and 0.86 [IQR: 0.78–0.98] for those starting BETER follow‐up later. In multivariable regression analysis, there was no difference in the EQ‐5D‐5L index score between the groups (prevalence risk ratio (PRR): 1.01, 95% CI: 0.79–1.29) (Table 2).

**TABLE 2:** Health‐related quality of life, knowledge about late effects, and distress among Hodgkin lymphoma survivors who started survivorship care follow‐up in 2013–2016, and among those who did not receive survivorship care until 2019–2024.^a^

	Survivors followed at a BETER clinic since 2013‐ 2016	Survivors without survivorship care until 2019–2024 = reference	Corresponding prevalence risk ratio (95% confidence interval)^b^
EQ‐5D‐5L index score (median [IQR])^c^	0.86 [0.78, 1.00]	0.85 [0.77, 1.00]	1.01 (0.79–1.29)
SF‐36 PCS (median [IQR])^d^, ^e^	50.3 [38.7, 55.9]	49.1 [37.6, 57.0]	0.98 (0.93–1.04)
SF‐36 MCS (median [IQR])^d^, ^e^	52.5 [42.5, 56.8]	52.0 [40.8, 57.7]	0.99 (0.93–1.05)
Knowledge questionnaire score (median [IQR])^d^	41.7 [26.7, 54.2]	45.6 [30.6, 57.8]	1.03 (0.92–1.18)
Modified CWS score (median [IQR])^f^	8.5 [7.0, 11.0]	9 [7.0–12.0]	0.98 (0.91–1.06)

Abbreviations: CWS, cancer worry scale; EQ‐5D‐5L, the five‐dimension/five‐level EuroQol questionnaire; MCS, mental component score; PCS, physical component score; SF‐36, the 36‐item Short Form Health Survey.

^a^ Results of the multivariable negative binomial regression analyses comparing the two study groups. All analyses were adjusted for sex, age at diagnosis, age at study inclusion, RT regimen, CT regimen, splenectomy/spleen RT, and education level. Shown scores were derived from the 20 imputed datasets. Scores from the complete case analysis are presented in Tables 3 and S5.

^b^ This prevalence risk ratio describes the difference in the score between the two study groups (BETER care since 2019–2024 = reference) given that the other variables in the model are held constant.

^c^ The scale of this score ranges from 0 to 1. Higher scores represent better performance. For one survivor with an extremely poor health condition, the EQ‐5D‐5L index score was below 0; this outlier value was set to 0 before the regression analysis.

^d^ The scale of this score ranges from 0 to 100. Higher scores represent better performance.

^e^ As we used Dutch normative data and Dutch factor scoring coefficients for the calculation of the scores on the SF‐36 PCS and MCS, a score of 50 represents the Dutch general population average [24, 30, 31].

^f^ The scale of this score ranges from 4 to 24. Lower scores represent better performance (less worries).

**FIGURE 3:**
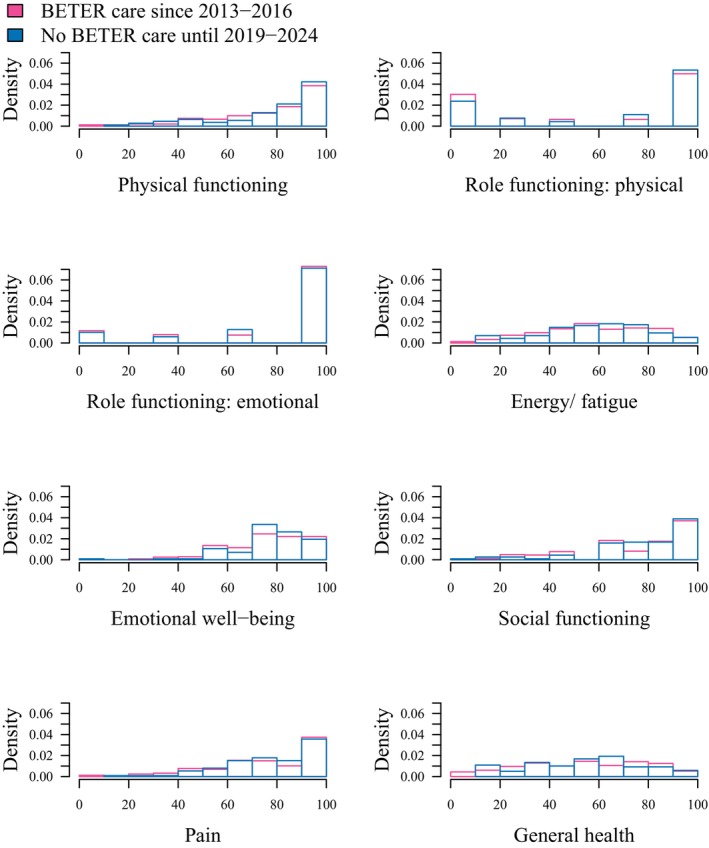
Histograms showing the density of the SF‐36 subscale scores for survivors followed at a BETER clinic since 2013‐2016 and for those without survivorship care until 2019–2024.

Scores on the SF‐36 subscales were similarly distributed among both groups. Moreover, median SF‐36 PCS and SF‐36 MCS were close to 50 in both groups (representing the average score in the Netherlands) (Figure 3 and Table 3).

**TABLE 3:** Health‐related quality of life among Hodgkin lymphoma survivors followed at a BETER survivorship care clinic since 2013–2016 and among those without survivorship care until 2019–2024.

	Survivors followed at a BETER clinic since 2013‐2016^a^	Survivors without survivorship care until 2019–2024^b^
EQ‐5D‐5L^a^, ^b^, ^c^		
Index score (median [IQR])	0.85 [0.77, 1.00]	0.86 [0.78, 0.98]
SF‐36^a^, ^d^		
Physical functioning (median [IQR])	85.0 [65.0, 95.0]	90.0 [75.0, 95.0]
Role limitations due to physical problems (median [IQR])	75 [0, 100]	100 [25.0, 100]
Role limitations due to emotional problems (median [IQR])	100 [66.7, 100]	100 [66.7, 100]
Energy/fatigue (median [IQR])	57.9 [42.1, 79.0]	63.2 [42.1, 73.7]
Emotional well‐being (median [IQR])	78.3 [60.9, 87.0]	79.3 [69.6, 87.0]
Social functioning (median [IQR])	87.5 [62.5, 100]	87.5 [62.5, 100]
Pain (median [IQR])	80.0 [67.5, 100]	88.8 [67.5, 100]
General health (median [IQR])	60.0 [35.0, 75.0]	60.0 [40.0, 70.0]
Physical component summary score (median [IQR])	49.6 [38.8, 62.5]	50.3 [38.5, 55.7]
Mental component summary score (median [IQR])	53.1 [42.8, 58.1]	52.3 [42.2, 56.7]

Abbreviations: EQ‐5D‐5L, the 5‐dimension/5‐level EuroQol; SF‐36, the 36‐item Short Form Health Survey.

^a^ Higher scores represent better functioning.

^b^ Index scores were missing for 2.8% of survivors followed at a BETER clinic since 2013‐2016 vs. 4.2% in those who started BETER follow‐up in 2019–2024. The complete cases are shown in this table.

^c^ Scores on the EQ‐5D‐5L subscales and overall visual analogue scale are presented in Figure 2.

^d^ Missing values for the SF‐36 in survivors followed at a BETER clinic since 2013‐2016 vs. those who started BETER follow‐up in 2019–2024: physical functioning (3.6% vs. 8.4%), role limitations due to physical problems (0% vs. 0.8%), role limitations due to emotional problems (14.7% vs. 0.8%), energy/fatigue (2.4% vs. 3.4%), emotional well‐being (2.8% vs. 5.0%), social functioning (2.4% vs. 5.0%), pain (2.0% vs. 5.9%), general health (1.2% vs. 0%), physical component summary score (20.3% vs. 14.3%), and mental component summary score (20.3% vs. 14.3%). The complete cases are shown in this table.

### 3.3 Knowledge About Late Effects

Knowledge about late adverse effects was better in survivors who started BETER follow‐up in 2013–2016 compared to those who started in 2019–2024 (median percentage correct answers 45.0% [IQR: 30.1, 56.9] vs. 40.8% [IQR: 26.7, 54.2] (Table S5)), but in multivariable analysis, there was no significant difference between the groups (PRR 1.03 (95% CI: 0.92–1.18, Table 2)). For female survivors, the median score on the knowledge questionnaire was 46.7% [IQR: 35.6, 58.3] in those who started BETER follow‐up in 2013–2016 compared to 43.3% [IQR: 30.0, 54.4] among survivors in the comparison group. For males, median scores were similar among the study groups (37.5% [IQR: 20.8–55.7] vs. 37.5% [IQR: 24.3, 51.7]). Multivariable analyses did not show significant differences in the knowledge scores for females, nor for males (Table S6).

### 3.4 Distress About Late Effects

Distress score was ~9 in both groups (the lowest possible score is 4/24), and the scores were also similar in the sex‐stratified subgroups (Table S5). In multivariable analysis, distress scores were not significantly different among the groups (PRR 0.98 (95% CI: 0.91–1.06, Table 2)), nor when analyzing females and males separately (Table S6).

### 3.5 Sensitivity Analyses

Results of the sensitivity analyses comparing survivors who had visited the BETER clinic at least twice since 2013–2016 to those who visited the clinic only once between 2019 and 2024 were consistent with the main analyses (Table S7).

### 3.6 Survivors' Experience With BETER Care

Overall, a quarter of survivors were not aware of the late effects of HL treatment before invitation to the BETER clinic. Two‐thirds of survivors felt that the advantages of more knowledge of late effects outweighed the disadvantages (more worries or anxiety). Seventy‐one percent felt they could talk about their (possible) worries during BETER visits. Almost 12% of survivors experienced the BETER visits as burdensome, and 80.5% of survivors perceived the visits as beneficial (Figure S1).

## 4 Discussion

In this large nationwide study with a high questionnaire response rate (72.1%), we found that HL survivors who regularly visited a BETER survivorship care clinic over a median follow‐up period of 6.5 years showed similar overall HRQoL compared with those only recently invited for BETER care. Moreover, no differences in distress levels or knowledge about late adverse effects were found between the groups. Importantly, 80% of survivors perceived the survivorship care visits as beneficial.

Existing evidence on the association between cancer survivorship care and HRQoL is limited. Most studies included a small sample of survivors and/or evaluated HRQoL only 6–14 months after the start of survivorship care [12, 13, 14, 17]. None of the earlier studies found increased HRQoL in those receiving survivorship care [12, 13, 14, 17]. For example, Viscuse et al. did not find a significant association between survivorship care and HRQoL or distress levels among 236 lymphoma survivors (among which 20 HL survivors) in the United States (response rate 65.2%) [12]. Similarly, Ford et al. observed no differences in HRQoL in adult childhood cancer survivors (mean age ~30 years) in the United States who did or did not attend a long‐term follow‐up clinic (*n* = 173, response rate unknown) [17].

The EQ‐5D‐5L index scores reported by our HL survivor population are very similar to those observed in the general population in the Netherlands (mean 0.87, standard deviation 0.17) [24]. As we used Dutch normative data and Dutch factor scoring coefficients for the calculation of the scores on the SF‐36 PCS and MCS, a score of 50 represents the Dutch general population average [20, 27, 28]. The observed medians of the PCS and MCS were close to 50 for both study groups. On a group level, HRQoL of the included HL survivors was very similar to that of the general population in the Netherlands, and there may be little potential for improvement. However, on an individual level, HRQoL among HL survivors may be reduced as a result of factors other than those addressed by the BETER survivorship care program. For example, many HL survivors experience persistent fatigue [30], for which there are only limited treatment options [10]. Other factors with substantial impact on HRQoL are socio‐economic factors such as employment status [31] and psychosocial stress such as depression or anxiety [32], for which the BETER program does not structurally provide specific interventions.

Based on existing evidence, we expected an association between participating in a survivorship care program and increased knowledge about late effects among HL survivors [12, 15, 18]. Viscuse et al. previously showed that lymphoma survivors attending a specialized survivorship clinic could better recall lifestyle advice and information on adverse effects compared to those not attending [12]. Moreover, Lindell et al. demonstrated increased knowledge of the risk of late effects among childhood cancer survivors receiving survivorship care compared to matched controls [18]. Our study, which was not primarily designed to study differences in knowledge about late adverse effects, could not confirm these findings. However, our comparison group had recently also started visiting a BETER clinic and visited the clinic more recently than the group followed at the BETER clinic from 2013 to 2016, which may have attenuated a possible difference.

Overall, survivors scored around 40%–45% correct on our exploratory (non‐validated) questionnaire assessing knowledge of late adverse effects. Detailed information about possible late effects is available on the BETER website, but survivors may not always be referred to this website, and/or information provision may not be well tailored to survivor preferences [33]. A mobile application including a personalized survivorship care plan and the use of social media to disseminate general patient information may be more effective [34, 35]. The majority of survivors reported that they felt that more knowledge of the late effects of their previous treatments outweighed (possible) worries about late adverse effects. The low distress levels in our study population support this fact. Our findings endorse the need for improved survivor education methods and survivor empowerment, as was demonstrated in other studies before [12, 30, 36, 37, 38].

### 4.1 Strengths and Limitations

A strength of our study is that, in contrast to other studies, the design allowed for the comparison of HRQoL between survivors who, due to logistic factors, were or were not invited for survivorship care over the past 6–10 years. These logistic factors include identification and tracking of eligible patients and setting up the local infrastructure for a survivorship clinic, including consulting specialists such as cardiologists [19]. In other studies, the comparison group consisted of survivors who had chosen not to attend a survivorship clinic, possibly related to their health status [12, 17]. Another major strength is that we used two different validated questionnaires to measure HRQoL, thereby decreasing the possibility of having missed possible differences in HRQoL between the study groups. A limitation of our study on patient‐reported outcomes is that selection bias may have been introduced, for example, if predominantly those with good HRQoL responded to the questionnaire, or if other responder characteristics were not representative of the source population. Our high response rate (72.1%) renders a substantial influence of such bias unlikely. We collected information on survivor and treatment characteristics of responders and non‐responders. We observed that responders were older than non‐responders, and therefore, we acknowledge that our study results may be less representative of the younger population of HL survivors.

## 5 Conclusion

Although we did not find an association between structured survivorship care and HRQoL among long‐term HL survivors, the vast majority of survivors perceived survivorship care as beneficial. In addition, knowledge about late effects among HL survivors is limited and needs more attention from caregivers. Our next step is to evaluate whether BETER survivorship care is associated with a decreased burden of disease attributed to late adverse effects of treatment [19]. Future research could include longitudinal measurements of HRQoL in survivors attending the BETER clinic in order to evaluate whether there is a change in HRQoL over time. Moreover, survivor preferences for risk communication and information provision should be scrutinized; a personalized survivorship care plan application might be valuable.

## Author Contributions

**Eline M. J. Lammers:** investigation, writing – original draft, writing – review and editing, data curation, project administration, visualization, formal analysis. **Annelies Nijdam:** conceptualization, supervision, writing – review and editing. **Michael Schaapveld:** writing – review and editing, methodology. **Lonneke V. van de Poll‐Franse:** writing – review and editing. **Cécile P. M. Janus:** writing – review and editing, resources. **Karin M. Aarsman:** resources, writing – review and editing. **Liane C. J. te Boome:** resources, writing – review and editing. **Nathalie B. Tomasoa:** writing – review and editing, resources. **Rinske S. Boersma:** resources, writing – review and editing. **Saskia E. Rademakers:** writing – review and editing, resources. **Floriske G. Stedema:** writing – review and editing, resources. **Lara H. Böhmer:** resources, writing – review and editing. **Marieke van den Berg:** resources, writing – review and editing. **Adriaantje C. Kroeze:** writing – review and editing, resources. **Leonie S. Strobbe:** resources, writing – review and editing. **Wendy Deenik:** writing – review and editing, resources. **Heleen S. de Lil:** resources, writing – review and editing. **Flora E. van Leeuwen:** conceptualization, writing – review and editing, supervision. **Berthe M. P. Aleman:** conceptualization, resources, writing – review and editing, supervision. **Josée M. Zijlstra:** resources, writing – review and editing, supervision.

## Ethics Statement

The INSIGHT study protocol was approved by the Institutional Review Board of The Netherlands Cancer Institute (IRB21‐115) and complied with local ethical approval guidelines at all 15 participating sites.

## Consent

Survivors who opted out of the use of their medical data for the INSIGHT study were excluded from the study.

## Conflicts of Interest

The authors declare no conflicts of interest.

## Supporting information


Data S1.

## Data Availability

The data underlying this article will be shared on reasonable request to the corresponding author.
