# Supplementary material for: Influence of Survivorship Care on Health‐Related Quality of Life, Knowledge of Late Effects, and Distress Levels Among Long‐Term Hodgkin Lymphoma Survivors
Source: Cancer Med. 2025 Aug 5;14(15):e71113. doi: 10.1002/cam4.71113 (PMC12322925; doi:10.1002/cam4.71113)
Supplement: Supplementary file 1 — Data S1. [file CAM4-14-e71113-s001.zip › Suppl Fig. 1.pdf]

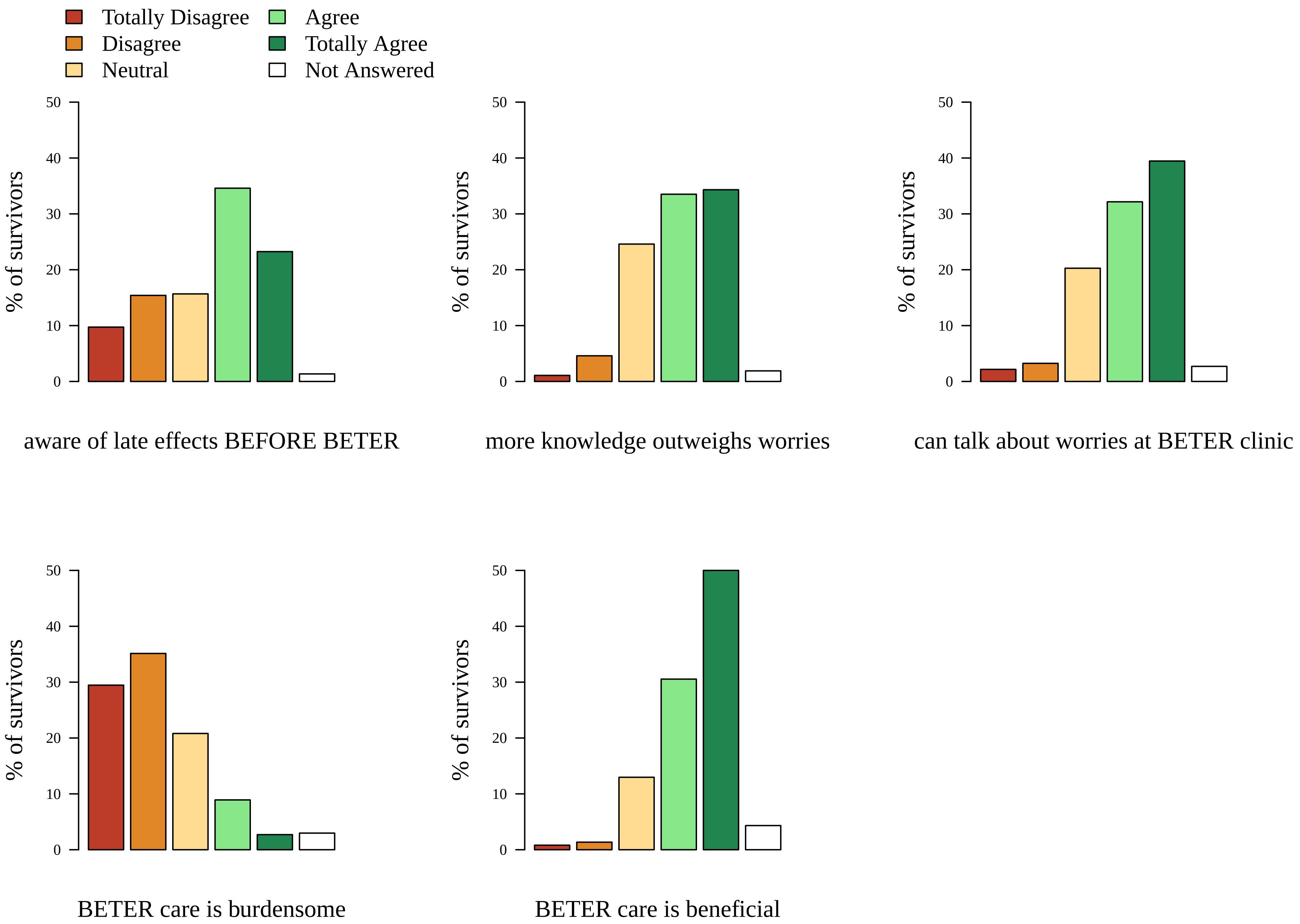
Totally Disagree
Disagree
Neutral
Agree
Totally Agree
Not Answered
% of survivors
0
10
20
30
40
50
aware of late effects BEFORE BETER
more knowledge outweighs worries
can talk about worries at BETER clinic
BETER care is burdensome
BETER care is beneficial
