# Supplementary material for: Influence of Survivorship Care on Health‐Related Quality of Life, Knowledge of Late Effects, and Distress Levels Among Long‐Term Hodgkin Lymphoma Survivors
Source: Cancer Med. 2025 Aug 5;14(15):e71113. doi: 10.1002/cam4.71113 (PMC12322925; doi:10.1002/cam4.71113)
Supplement: Supplementary file 1 — Data S1. [file CAM4-14-e71113-s001.zip › Supplement_QoL_INSIGHT_Cancer.docx]

**Supplementary material**

| **Supplementary Table 1:** Questionnaire on knowledge about late effects (original questions were in Dutch)^1^ | | | |
| --- | --- | --- | --- |
| *Question/ statement* | *Answer options* | *Correct answer(s)* | *Survivor answers and assigned points* |
| 1. People who were irradiated to the neck have high risk of: | 1. Hyperthyroidism 2. Hypothyroidism 3. Both of the above answers are correct 4. Both of the above answers are incorrect | C | A: <1% (1.5 point)  B: 31.3% (1.5 point)  C: 22.4% (3 points)  D: 3.2% (0 points)  I don’t know: 41.4% (0 points)  Missing: 1% |
| 1. Heart diseases (e.g. heart failure or valve dysfunctions) after treatment for Hodgkin lymphoma are associated with: | 1. Mediastinal radiotherapy 2. Treatment with anthracycline-based chemotherapy (e.g. as part of BEACOPP or ABVD chemotherapy regimen) 3. Both of the above answers are correct 4. Both of the above answers are incorrect | C | A: 27.3% (1.5 point)  B: 13.5% (1.5 point)  C: 27.6% (3 points)  D: 1% (0 points)  I don’t know: 29.5% (0 points)  Missing: 1% |
| 1. A person with hypertension after Hodgkin lymphoma treatment has a similar risk of development of cardiovascular diseases as a person not treated for Hodgkin lymphoma. | 1. Correct 2. Incorrect | B | A: 20.8% (0 points)  B: 20.3% (3 points)  I don’t know: 55.4% (0 points)  Missing: 3.5% |
| 1. Fatigue after Hodgkin lymphoma only occurs in the first five years after treatment. | 1. Correct 2. Incorrect | B | A: 6.5% (0 points)  B: 69.5% (3 points)  I don’t know: 21.1% (0 points)  Missing: 3.0% |
| 1. Certain types of chemotherapy increase the risk of infertility in: | 1. Men 2. Women 3. Men and Women | C | A: 11.6% (0 points)  B: 10.8% (0 points)  C: 46.2% (3 points)  I don’t know: 22.7% (0 points)  Missing: 3.2% |
| 1. Smoking increases the risk of several diseases in the general population. After Hodgkin lymphoma treatment the unfavorable effect of smoking on health is much bigger. The risk of the following diseases is increased in a Hodgkin lymphoma survivor who smokes:^2^ | 1. Acute leukemia 2. Cardiovascular diseases, e.g. myocardial infarction or heart failure 3. Lung diseases | A, B and C | A, B and C selected: 8.1% (3 points)  A selected: 8.9% (1 point)  B selected: 44.9% (1 point)  C selected: 51.6% (1 point)  I don’t know: 39.7% (0 points)  Missing: 9.6% |
| 1. After Hodgkin lymphoma some mental and neurological complaints occur more often, these include:^2^ | 1. Depressive complaints, e.g. gloom and apathy 2. Cognitive problems, e.g. problems with concentration and attention 3. Psychotic complaints, e.g. delusions and hallucinations | A and B | A and B selected: 36.8% (3 points)  A selected: 49.2% (1.5 point)  B selected: 51.9% (1.5 point)  C selected: 3.2% (0 points)  I don’t know: 34.3% (0 points)  Missing: 9.6% |
| 1. After Hodgkin lymphoma treatment some physical complaints occur more often. Which complaints are these?^2^ | 1. Skin complaints, e.g. rash or brown discoloration 2. Migraine 3. Neuropathy | A and C | A and C selected: 16.2% (3 points)  A selected: 29.5% (1.5 point)  B selected: 11.1% (0 points)  C selected: 50.5% (1.5 point)  I don’t know: 35.9% (0 points)  Missing: 9.5% |
| 1. Some treatments for Hodgkin lymphoma contain prednisone, a medicine that increases the risk of osteoporosis. The risk of osteoporosis can be decreased by: | 1. Sufficient intake of calcium and vitamin D, and/or absorption of vitamin D through sunlight 2. Regular practice of sports such as running, dancing or jump rope 3. Both of the above answers are correct 4. Both of the above answers are incorrect | C | A: 21.1% (1.5 point)  B: 3.2% (1.5 point)  C: 46.2% (3 points)  D: <1% (0 points)  I don’t know: 24.3% (0 points)  Missing: 4.6% |
| 1. A person is treated for Hodgkin lymphoma with radiotherapy to the neck. What is the chance of development of hypothyroidism during his/her entire life? | Survivors had to estimate a percentage between 0 and 100% on a visual scale, and answers of ±10% from exact answer were counted as correct. | [36-56%] | Correct answer: 32.4% (3 points)  Missing: 7.6% |
| 1. A person irradiated to the spleen is at higher risk of: | 1. Infections with certain bacteria that cause pneumonia or meningitis 2. Infection after a dog or cat bite 3. Both of the above answers are correct 4. Both of the above answers are incorrect | C | A: 10.3% (1.5 point)  B: <1% (1.5 point)  C: 15.4% (3 points)  D: 1.4% (0 points)  I don’t know: 66.2% (0 points)  Missing: 6.5% |
| 1. People who had a splenectomy or who were irradiated to the spleen have to take antibiotics immediately in case of fever, without permission of a doctor. | 1. Correct 2. Incorrect | A | A: 15.7% (3 points)  B: 12.7% (0 points)  I don’t know: 65.1% (0 points)  Missing: 6.5% |
| 1. Women who retained their menstrual cycle after Hodgkin lymphoma treatment can enter menopause prematurely (before the age of 40 years).^3^ | 1. Correct 2. Incorrect | A | A: 57.9% (3 points)  B: 4.6% (0 points)  I don’t know: 29.7% (0 points)  Missing: 7.8% |
| 1. Women irradiated to the chest before the age of 40 years have an increased risk of breast cancer. In order to detect breast cancer in an early stage these women are advised to yearly undergo a mammography and a MRI of the breasts.^3^ | 1. Correct 2. Incorrect | A | A: 68.2% (3 points)  B: 3.1% (0 points)  I don’t know: 21.5% (0 points)  Missing: 7.2% |
| 1. What do you think the chance of development of breast cancer is for a woman treated with mantle field irradiation in the 30 years after treatment?^3^ | Survivors had to estimate a percentage between 0 and 100% on a visual scale, and answers of ±10% from exact answer were counted as correct. | [10-30%] | Correct: 64.2% (3 points)  Missing: 9.2% |
| ^1^ Original questions were in lay language and contained extra explanations. For multiple-choice questions there was an option “I don’t know” to distinguish between not knowing the answer and forgetting to fill in the question.  ^2^ For this question the participant was informed that multiple answers are correct. Points were not diminished when the survivor had selected a wrong answer for question 7 and 8.  ^3^ This question only applied to females.  *Abbreviations: ABVD = Adriamycin (Doxorubicin), Bleomycin, Vinblastine, and Dacarbazine, BEACOPP = Bleomycin, Etoposide, Adriamycin (Doxorubicin), Cyclophosphamide, Vincristine, Procarbazine, and Prednisone.* | | | |

| **Supplementary Table 2:** Modified version of the Cancer Worry Scale (1) | |
| --- | --- |
| *Question* | *Answer options* |
| 1. How often have you thought about your chance of development or worsening of late adverse effects of Hodgkin lymphoma treatment during the past month? | 1. Almost never 2. Sometimes 3. Often 4. Almost always |
| 1. How often have your thoughts about your chance of development or worsening of late adverse effects of Hodgkin lymphoma treatment influenced your mood during the past month? | 1. Almost never 2. Sometimes 3. Often 4. Almost always |
| 1. How often have your thoughts about your chance of development or worsening of late adverse effects of Hodgkin lymphoma treatment interfered with your ability to do daily activities during the past month? | 1. Almost never 2. Sometimes 3. Often 4. Almost always |
| 1. How concerned are you about the possibility that you, as a result of the treatment, develop a different type of cancer (again)? | 1. Not at all 2. A little 3. Quite 4. Very much |
| 1. How often did you, during the past month, worry about your chance of development or worsening of late adverse effects of Hodgkin lymphoma treatment? | 1. Almost never 2. Sometimes 3. Often 4. Almost always |
| 1. How much of a problem is this worry about your chance of development or worsening of late adverse effects of Hodgkin lymphoma treatment? | 1. Not at all 2. A little 3. Quite 4. Very much |

| **Supplementary Table 3:** Questions on survivor experience with BETER care | |
| --- | --- |
| *Question* | *Answer options* |
| 1. I was, before I received the invitation letter for BETER care, aware of possible long-term effects of Hodgkin lymphoma treatment. | 1. Totally agree 2. Agree 3. Neutral 4. Disagree 5. Totally disagree |
| 1. The advantages of more knowledge of late effects of Hodgkin lymphoma treatment outweigh the disadvantages of more knowledge (more worries or anxiety). | 1. Totally agree 2. Agree 3. Neutral 4. Disagree 5. Totally disagree |
| 1. During an appointment with my BETER caregiver, I can talk about matters that I am concerned or worried about. | 1. Totally agree 2. Agree 3. Neutral 4. Disagree 5. Totally disagree |
| 1. I find the BETER visits burdensome. | 1. Totally agree 2. Agree 3. Neutral 4. Disagree 5. Totally disagree |
| 1. I find the BETER visits beneficial. | 1. Totally agree 2. Agree 3. Neutral 4. Disagree 5. Totally disagree |

| **Supplementary Table 4:** Characteristics of responders and non-responders of the INSIGHT study questionnaire for all attenders of BETER care (i.e. both study groups together) | | | | |
| --- | --- | --- | --- | --- |
|  | | | **Responders** | **Non-responders** |
| n (%) | | | 370 (72.1) | 143 (27.9) |
| Female sex (%) | | | 195 (52.7) | 83 (58.0) |
| Age at HL diagnosis (years) (median [IQR]) | | | 25.9 [21.2, 33.1] | 25.0 [20.4, 30.2] |
| Age at HL diagnosis (years) (cat.) (%) | 10-19 | | 67 (18.1) | 38 (26.6) |
|  | 20-29 | | 176 (47.6) | 68 (47.6) |
|  | 30-39 | | 87 (23.5) | 30 (21.0) |
|  | 40-49 | | 34 (9.2) | 7 (4.9) |
|  | 50-59 | | 6 (1.6) | 0 (0.0) |
| Year of HL diagnosis (cat.) (%) | 1971-1980 | | 22 (5.9) | 3 (2.1) |
|  | 1981-1990 | | 75 (20.3) | 16 (11.2) |
|  | 1991-2000 | | 135 (36.5) | 47 (32.9) |
|  | 2001-2011 | | 138 (37.3) | 77 (53.8) |
| Age at study invitation (years) (median [IQR]) | | | 55.8 [47.5, 62.4] | 48.8 [42.3, 54.9] |
| Age at study invitation (years) (cat.) (%) | | 27-34 | 8 (2.2) | 6 (4.2) |
|  |  | 35-44 | 67 (18.1) | 42 (29.4) |
|  |  | 45-54 | 99 (26.8) | 60 (42.0) |
|  |  | 55-64 | 147 (39.7) | 27 (18.9) |
|  |  | 65-71 | 49 (13.2) | 8 (5.6) |
| Time between diagnosis and study invitation (years) (median [IQR]) | | | 24.8 [18.8, 32.4] | 21.1 [17.4, 28.3] |
| Time between last BETER visit and survey invitation (years) median [IQR]) | | | 1.06 [0.32, 3.52] | 1.08 [0.29, 3.76] |
| Age at first BETER visit (years) (median [IQR]) | | | 50.3 [41.5, 56.9] | 44.0 [37.5, 51.4] |
| Age at first BETER visit (cat.) (%) | 20-29 | | 15 (4.1) | 12 (8.4) |
|  | 30-39 | | 65 (17.6) | 35 (24.5) |
|  | 40-49 | | 98 (26.5) | 54 (37.8) |
|  | 50-59 | | 143 (38.6) | 36 (25.2) |
|  | 60-70 | | 49 (13.2) | 6 (4.2) |
| Time between diagnosis and first BETER visit (years) (median [IQR]) | | | 20.3 [13.3, 27.9] | 18.1 [10.8, 23.3] |
| RT regimen (%) | RT to full mantle field | | 92 (24.9) | 22 (15.4) |
|  | mediastinal RT but no full mantle field | | 160 (43.2) | 65 (45.5) |
|  | RT to neck but no mediastinal nor full mantle field RT | | 36 (9.7) | 17 (11.9) |
|  | RT to other fields or no RT | | 82 ( 22.2) | 39 (27.3) |
| CT regimen (%) | containing both anthracyclines and procarbazine | | 119 (32.2) | 51 (35.7) |
|  | containing anthracyclines but no procarbazine | | 157 (42.4) | 69 (48.3) |
|  | containing procarbazine but no anthracyclines | | 56 (15.1) | 15 (10.5) |
|  | no CT | | 56 (15.1) | 15 (10.5) |
| Splenectomy or spleen RT (%) | | | 94 (25.4) | 26 (18.2) |
| SES score^1^ (median [IQR]) | | | 0.17 [0.01, 0.26] | 0.12 [-0.03, 0.24] |
| ^1^SES score was derived from survivors’ zip codes at study inclusion using Statistics Netherlands data (version 2021, excluding students) (2). The SES score per zip code area is calculated by Statistics Netherlands based on: welfare level (i.e. income and assets), education, and recent labor participation. A score of 0 represents the average score in the Netherlands.  *Abbreviations: CT = chemotherapy, HL = Hodgkin lymphoma, RT = radiotherapy, SES = socio-economic status* | | | | |

| **Supplementary Table 5:** Total scores on the knowledge questionnaire and modified Cancer Worry Scale | | | | | | |
| --- | --- | --- | --- | --- | --- | --- |
|  | **Overall** | | **Females** | | **Males** | |
|  | *Survivors followed at a BETER clinic since 2013-2016* | *Survivors without survivorship care until 2019-2024* | *Survivors followed at a BETER clinic since 2013-2016* | *Survivors without survivorship care until 2019-2024* | *Survivors followed at a BETER clinic since 2013-2016* | *Survivors without survivorship care until 2019-2024* |
| Percentage correct on knowledge questionnaire (median [IQR]) (n)^1^ | 45.0 [30.1,56.9] (n=218) | 40.8 [26.7,54.2]  (n=100) | 46.7 [35.6,58.3] (n=115) | 43.3 [30.0,54.4] (n=48) | 37.5 [20.8,55.7]  (n=103) | 37.5 [24.3,51.7]  (n=52) |
| Score on the modified CWS (median [IQR]) (n)^2^ | 9 [7,12]  (n=246) | 8.5 [7,11]  (n=112) | 9 [8,12]  (n=134) | 9 [7.8,11]  (n=56) | 8 [7,11]  (n=112) | 8 [7,11]  (n=56) |
| ^1^ Scores on the knowledge questionnaire could not be calculated due to missing values for 15.4% of female survivors followed at a BETER clinic since 2013-2016 and for 18.6% of females in the comparison group, for 10.4% of males followed at a BETER clinic since 2013-2016 and for 13.3% of males in the comparison group. Overall, 14.1% had of survivors a missing knowledge score, for the missing percentage per question see *Supplementary Table 1*. The complete cases are shown in this table.  ^2^ Scores on the 6-item CWS may range from 4 to 24 (1-4 points per item). CWS scores could not be calculated due to missing values for 1.5% of females followed at a BETER clinic since 2013-2016 and for 5.1% of females in the comparison group, for 2.6% of males followed at a BETER clinic since 2013-2016 and for 6.7% of females in the comparison group.  *Abbreviation: CWS = Cancer Worry Scale* | | | | | | |

| **Supplementary Table 6:** *Subgroup analyses*: distress and knowledge in survivors followed at a BETER clinic since 2013-2016 compared to survivors not receiving survivorship care until 2019-2024, stratified by sex | | | | |
| --- | --- | --- | --- | --- |
|  | *Males* | | *Females* | |
|  | **Prevalence Risk Ratio^2^** | **95% Confidence Interval** | **Prevalence Risk Ratio^2^** | **95% Confidence Interval** |
| Knowledge questionnaire score^3^ | 1.01 | 0.82-1.25 | 1.06 | 0.92-1.22 |
| Modified CWS score^4^ | 0.96 | 0.85-1.08 | 0.98 | 0.89-1.09 |
| ^1^ These analyses were controlled for age at diagnosis, age at study inclusion, RT regimen, CT regimen, splenectomy/spleen RT, and education level.  ^2^ This prevalence risk ratio describes the difference in the score between the study groups (BETER care since 2019-2024 = reference), given that the other variables in the model are held constant.  ^3^ Higher scores represent better performance.  ^4^ Lower scores represent better performance (less worries).  *Abbreviations: CWS = cancer worry scale, EQ-5D-5L = the 5 dimension/5-level EuroQol questionnaire, MCS = mental component score, PCS = physical component score, SF-36 = the 36-item Short Form Health Survey.* | | | | |

| **Supplementary Table 7:** *Sensitivity analyses*: survivors who visited BETER clinic at least twice since 2013-2016 (n=201) vs. survivors who visited the BETER clinic once since 2019-2024 (n=92)^1^ | | |
| --- | --- | --- |
|  | **Prevalence Risk Ratio^2^** | **95% Confidence Interval** |
| EQ-5D-5L index score^3,4^ | 1.03 | 0.77-1.36 |
| SF-36 PCS^3^ | 1.01 | 0.95-1.08 |
| SF-36 MCS^3^ | 1.01 | 0.94-1.08 |
| Knowledge questionnaire score^3^ | 1.03 | 0.90-1.17 |
| Modified CWS score^5^ | 0.96 | 0.88-1.05 |
| ^1^ These analyses were controlled for sex, age at diagnosis, age at study inclusion, RT regimen, CT regimen, splenectomy/spleen RT, and education level.  ^2^ This prevalence risk ratio describes the difference in the score between the study groups (BETER care since 2019-2024 = reference), given that the other variables in the model are held constant.  ^3^ Higher scores represent better performance.  ^4^ For one survivor with an extremely poor health condition the EQ-5D-5L index score was below 0, this outlier value was set to 0 before the regression analysis.  ^5^ Lower scores represent better performance (less worries).  *Abbreviations: CWS = cancer worry scale, EQ-5D-5L = the 5 dimension/5-level EuroQol questionnaire, MCS = mental component score, PCS = physical component score, SF-36 = the 36-item Short Form Health Survey.* | | |


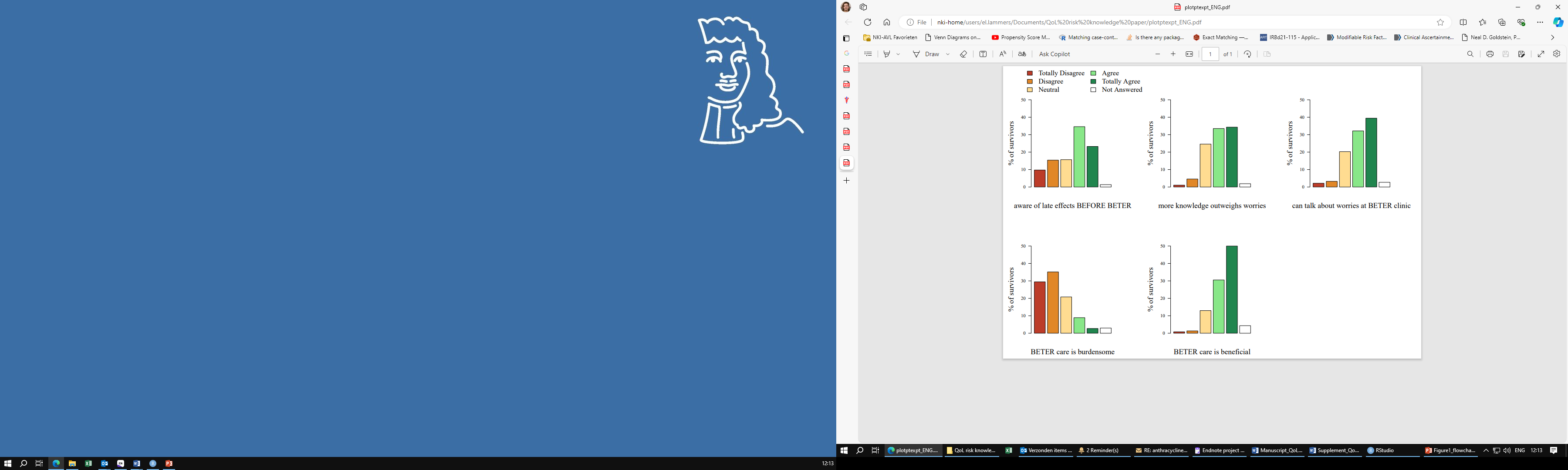


**Supplementary Figure 1:** Experiences with BETER care (all responders in the entire study (n = 370)).

**References**

1. Custers JAE, Kwakkenbos L, van de Wal M, Prins JB, Thewes B. Re-validation and screening capacity of the 6-item version of the Cancer Worry Scale. Psychooncology. 2018;27(11):2609-15.

2. Statistics Netherlands. Socioeconomic status based on zip code, excluding students. 2021. https://www.cbs.nl/nl-nl/maatwerk/2023/33/sociaal-economische-status-per-postcode-2020-en-2021. Accessed 22 August 2024.
